# Severe Early-Onset Withdrawal Following Intentional Use of Mitragynine Pseudoindoxyl: A Case Report and Emerging Clinical Considerations

**DOI:** 10.7759/cureus.105224

**Published:** 2026-03-14

**Authors:** Corneliu N Stanciu

**Affiliations:** 1 Psychiatry, Geisel School of Medicine, Concord, USA

**Keywords:** 7-hydroxymitragynine, addiction psychiatry, kratom, mitragynine pseudoindoxyl, opioids

## Abstract

Kratom is increasingly used in the United States for pain, mood, anxiety, and opioid substitution. Traditional kratom produces mild, delayed withdrawal, but semisynthetic derivatives such as mitragynine pseudoindoxyl (MP) are highly potent μ-opioid receptor agonists with δ-opioid receptor antagonism, leading to rapid-onset, full-spectrum opioid-like withdrawal. We describe a 34-year-old man with long-term remission from heroin use disorder who presented with suicidal ideation and severe opioid-like withdrawal despite no recent opioid use. He progressed from powdered kratom to 7-hydroxymitragynine tablets and then MP tablets, averaging nine 20 mg doses daily, including nocturnal use. On presentation, he exhibited chills, tremors, diaphoresis, gastrointestinal distress, restlessness, with a Clinical Opiate Withdrawal Scale score of 31, blood pressure 168/107 mmHg, and heart rate 115 bpm. Supportive management with clonidine, hydroxyzine, gabapentin, loperamide, and antiemetics over 72-96 hours led to symptom resolution. Naltrexone, including a Vivitrol injection, was initiated without precipitating withdrawal, and the patient remained abstinent at one-month follow-up, despite a brief noneuphoric lapse. This case highlights MP as an emerging high-potency kratom derivative that poses unique diagnostic and management challenges. Clinicians should recognize its severe withdrawal profile, the limitations of standard toxicology, and the potential role of naltrexone in relapse prevention once an opioid-free interval is established.

## Introduction

Kratom (Mitragyna speciosa) has gained substantial popularity in the United States as a self-management tool for pain, anxiety, mood symptoms, and as an opioid replacement [1,2]. Although kratom has been used for centuries in Southeast Asia with relatively low concern for severe toxicities and liabilities, the contemporary U.S. market has evolved in ways that diverge substantially from traditional botanical preparations [3]. To better characterize this transformation, we introduce a conceptual “three waves” framework describing the progression of kratom products in the United States. The first wave consisted primarily of ground leaf powders with alkaloid profiles comparable to those of the natural plant. The second wave emerged with the adoption of solvent-based extraction techniques designed to concentrate specific alkaloids naturally present in kratom [4]. The current third wave represents a marked departure from botanical kratom: manufacturers are now producing laboratory-derived compounds by chemically modifying kratom’s primary alkaloid, mitragynine (MG), into isolated, highly potent derivatives. These products are frequently formulated into pills or concentrated preparations and marketed as “kratom,” despite being chemically and pharmacologically distinct from traditional plant material [5].

MG is a partial μ-opioid receptor (MOR) agonist with atypical G-protein-biased signaling. Withdrawal from kratom containing traditional MG concentrations typically has a delayed onset and a milder clinical course compared to classical opioids. Hepatic CYP3A metabolism converts a portion of MG to 7-hydroxymitragynine (7-OHMG), and small amounts form naturally in the dried leaf. Further oxidative transformation yields mitragynine pseudoindoxyl (MP), a semisynthetic derivative that is not naturally present in kratom [6]. In receptor binding assays, MG demonstrates relatively low MOR affinity (Ki ~709 nM range), whereas 7-OHMG exhibits substantially higher affinity (Ki ~78 nM). MP shows even greater MOR potency and intrinsic efficacy (Ki ~1.5 nM) and near-full agonist activity in preclinical systems, exceeding morphine in functional assays [7,8]. In addition, MP exhibits δ-opioid receptor (DOR) antagonism. Although MG and 7-OHMG also demonstrate DOR antagonism, MP is distinguished by its markedly greater MOR intrinsic efficacy, approaching full agonism in preclinical systems. The combination of high MOR activation with concurrent DOR blockade may alter downstream signaling dynamics. This pharmacological profile may contribute to dysphoria and unusually intense withdrawal syndromes [9].

Although MP was initially identified only as a potential adulterant in some kratom preparations, it is now being intentionally sold as a stand-alone product or included in highly concentrated “extract” products labeled as kratom. In many cases, vendors explicitly label these products as containing MP or other high-potency alkaloids and may specify the dose per unit; however, labeling practices are inconsistent across manufacturers, and product composition is not independently verified. These laboratory-derived preparations have dramatically different pharmacological effects and risk profiles relative to traditional plant material. Labeling, when available, does not list MP amounts contained in the product. Clinical recognition is challenging because standard toxicology screens do not detect MP, leaving clinicians to rely on patient history and symptomatology. This case illustrates the presentation of MP-associated withdrawal, which appears more rapid in onset, more severe, and more consistent with full-spectrum opioid withdrawal than withdrawal from traditional kratom products. Awareness of these distinctions is increasingly important as clinicians encounter patients exposed to manipulated, high-potency “kratom” analogs. Notably, in this case, concurrent naltrexone use appeared to blunt the expected euphoric effects of MP, providing additional insight into its opioid-mediated mechanism of action.

## Case presentation

A 34-year-old man with a history of depression, yet off psychotropics, presented to the emergency department with suicidal ideation and depressive symptoms and was subsequently admitted to the inpatient psychiatry unit. On admission, he endorsed no recent opioid exposure, and this was confirmed by a negative urine toxicology screen. This was a routine hospital urine immunoassay panel, which detects natural opioids as well as common synthetic opioids such as oxycodone, buprenorphine, methadone, and fentanyl. He also reported a history of remission from opioid use disorder for six years. Despite this, he was experiencing chills, cold flashes, restlessness, piloerection, diaphoresis, mild body aches, and nausea. Over the next two hours, these symptoms evolved into severe body aches, tremors, pronounced restlessness, diarrhea requiring frequent bathroom use, and two episodes of vomiting. Vital signs demonstrated blood pressure of 168/107 and a heart rate of 115. Routine laboratory studies, including electrolytes, kidney and liver function tests, were unremarkable. The patient denied any illicit drug use but reported regular "kratom" consumption, noting that similar withdrawal-like symptoms occurred at home if dosing intervals exceeded five to six hours. He had last used the product seven hours before the presentation.

The patient reported initiating kratom use five years before, approximately one year after achieving heroin remission, initially using powdered kratom (one teaspoon in the morning, two in the evening), often without experiencing withdrawal for several weeks. About one year ago, he began using “7-hydroxy” (7-OHMG) tablets, which he perceived as more potent for mood and euphoria. Within one month, his dose escalated from two 15 mg tablets daily to 30 mg three to four times daily, and three months before admission, he switched to a 65 mg tablet formulation, taking four to six times daily, including nocturnal doses. He reported mild chills and discomfort upon awakening when doses were delayed.

Approximately three months before this admission, he began experimenting with MP tablets, initially taking two 8 mg tablets, which he found more potent than 7‑OH. He subsequently transitioned to a different brand of MP tablets, taking approximately 20 mg per dose, averaging nine doses per 24 hours, including one or two nocturnal doses. This pattern caused marital stress due to financial strain, decreased productivity at work, lethargy, and disengagement at home. The patient also reported involvement in a recent motor vehicle accident while using MP. Attempts to reduce dose or frequency were unsuccessful due to extreme discomfort described as “crawling out of my skin.” Despite awareness of potential risks from online forums, he was unable to stop using and expressed interest in “getting off these things” while inpatient.

The patient’s Clinical Opiate Withdrawal Scale (COWS) score was 31 [10]. He was treated with supportive and symptomatic measures, including clonidine 0.2 mg every six hours, hydroxyzine 50 mg every six hours as needed for anxiety, Compazine 5 mg every six hours as needed for nausea, loperamide 2 mg every four hours as needed, and gabapentin 600 mg four times daily. His symptoms peaked over the first 24 hours and gradually improved over 72-96 hours. COWS scores remained elevated in the moderately severe range for the first 12 hours, with continued need for all pro re nata (PRNs) during the first two days. By day 2, the COWS score decreased to 10, and by day 3, it was 8, with minimal need for PRNs.

By day 5, the patient was symptom-free. Paroxetine was initiated based on prior response for depressive symptoms, and a long-term sobriety plan was discussed, including naltrexone therapy. On day 6, an oral naltrexone test dose was administered without precipitating withdrawal, followed by a naltrexone depot (Vivitrol) injection on day 7 before discharge. He was referred to outpatient addiction services for ongoing monitoring and support.

**Figure 1:**
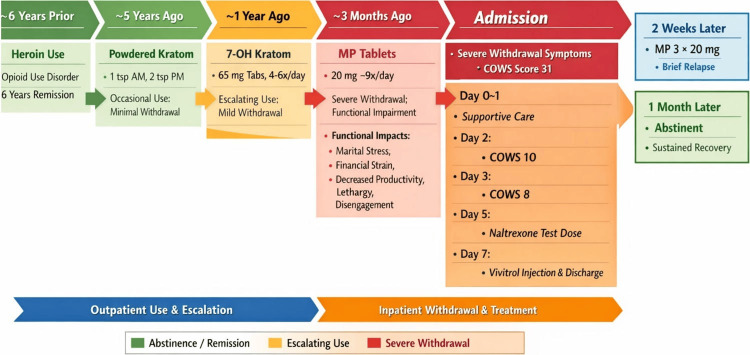
Chronology of kratom use, addiction progression, withdrawal, and maintenance treatment COWS: Clinical Opiate Withdrawal Scale

Two weeks after discharge, following a stressful event, the patient ingested three 20 mg MP tablets remaining at home. He reported fatigue but no euphoria. At one-month follow-up, he remained abstinent from MP and continued engagement with outpatient addiction support. A chronology of the patient's kratom use, progression, withdrawal, and management is depicted in Figure 1.

## Discussion

MP is now commercially available both as a stand-alone compound and as a concentrated ingredient within some products marketed as “kratom.” This development marks a significant shift within the unregulated kratom marketplace, reflecting the emergence of semisynthetic, high-potency derivatives that differ substantially from traditional botanical preparations [11]. Compared with MG, MP demonstrates markedly greater potency, high MOR occupancy, and DOR antagonism. These properties offer a mechanistic explanation for the rapid onset, severity, and full-spectrum opioid-like withdrawal observed, features not previously typical of classic kratom withdrawal. However, confounding factors should be acknowledged: the patient had been using 260-390 mg/day of 7-OHMG with established interdose withdrawal prior to transitioning to 180 mg/day of MP. Without complete characterization of 7-OHMG vs. MP pharmacology, including oral bioavailability and blood-brain barrier penetration in humans, it is unclear whether 180 mg/day of MP truly represents greater total opioid exposure than the prior 7-OHMG regimen. Thus, the severity of withdrawal may reflect cumulative dependence across his kratom alkaloid use rather than being solely attributable to MP’s pharmacologic profile.

These pharmacologic distinctions underscore that MP withdrawal represents a fundamentally different clinical entity. Whereas traditional kratom withdrawal is typically delayed and comparatively mild, MP’s pharmacokinetic characteristics, including rapid central nervous system penetration and high receptor occupancy at nanomolar concentrations in preclinical models, result in an earlier onset and more intense opioid-like effects than seen with botanical kratom alkaloids. In this respect, the clinical syndrome of MP withdrawal may more closely resemble that of short-acting prescription opioids, which are characterized by rapid absorption and a relatively brief elimination half-life, leading to earlier and more pronounced withdrawal symptom onset. Such presentation necessitates closer monitoring and supportive care aligned with established opioid withdrawal management protocols [12]. This includes hydration, autonomic stabilization, antiemetics, symptomatic medications, and, when clinically appropriate, opioid agonist therapy [13].

Recognition is further complicated by the fact that routine urine drug screens do not detect MP or related semisynthetic alkaloids. At present, detection is limited to specialized mass spectrometry assays available through select research laboratories. This creates a diagnostic challenge and reinforces the need for clinicians to obtain a detailed substance use history, including inquiry about online vendors, “research chemicals,” and products advertised as “extracts,” “enhanced,” or “potent kratom derivatives.”

The growing online presence of MP, often marketed explicitly for its strong psychoactive and opioid-like effects, raises substantial public health and harm-reduction concerns. Some users intentionally seek more potent alternatives to kratom, while others may unknowingly consume MP in adulterated or mislabeled products. Early recognition of MP-related withdrawal is, therefore, essential to prevent unnecessary diagnostic workups, avoid misclassification as primary opioid use disorder, and guide appropriate supportive care.

The regulatory landscape surrounding kratom alkaloids and their derivatives is rapidly evolving. In July 2025, the FDA recommended federal scheduling of 7-OHMG due to concerns regarding potency and opioid-like pharmacology. While MP and related semisynthetic derivatives are not uniformly scheduled, increasing regulatory scrutiny of high-potency kratom constituents may alter product availability and marketplace behavior. Awareness of this shifting context is important, as changes in regulation can influence formulation practices, adulteration patterns, and patient exposure risk.

Clinicians should incorporate MP and other semisynthetic kratom derivatives into the differential diagnosis when encountering patients with early-onset, intense opioid-like withdrawal despite reporting only “kratom” use. These compounds are not detected on routine toxicology screens; confirmatory testing typically requires specialized mass spectrometry-based assays that are not widely accessible, making clinical recognition essential. Related MG analogs may present with similar intoxication and withdrawal syndromes. Awareness of these emerging products may help avert iatrogenic complications and ensure accurate, mechanism-informed management. Reporting suspected cases to poison centers, public health entities, or surveillance programs is critical to improving detection and monitoring of this evolving class of psychoactive substances.

An additional clinically relevant observation in this case was the absence of euphoria when the patient ingested three 20 mg MP tablets approximately two weeks after receiving extended-release naltrexone. Despite MP’s potent MOR agonism, the lack of subjective opioid effects is pharmacologically plausible. Naltrexone exhibits exceptionally high affinity for the MOR, with reported Ki values in the subnanomolar range (approximately 0.11 nM), indicating very tight receptor binding and slow dissociation [9]. As a competitive antagonist, naltrexone can, therefore, maintain substantial receptor occupancy and prevent agonist-mediated signaling even when potent opioids are subsequently introduced. The patient’s experience suggests that extended-release naltrexone may effectively block the reinforcing effects of MP in a manner analogous to other opioid agonists. Although this observation derives from a single case and the precise receptor pharmacodynamics of MP in humans remain incompletely characterized, it raises the possibility that antagonist therapy may represent a viable relapse-prevention strategy for individuals dependent on high-potency kratom derivatives.

However, this pharmacologic property also carries clinical risk. Because MP acts as a potent MOR agonist, administration of naltrexone in an individual actively using MP could precipitate severe withdrawal. As with other opioids, initiation of antagonist therapy should, therefore, only occur after a confirmed opioid-free interval and careful clinical assessment to minimize the risk of precipitated withdrawal.

## Conclusions

MP represents a highly potent, semisynthetic kratom derivative whose clinical effects and withdrawal profile differ substantially from traditional botanical kratom. As availability expands through both online vendors and brick-and-mortar retail settings (e.g., smoke shops, vape shops, and gas stations), intentional use appears to be increasing, and some individuals may unknowingly encounter MP in adulterated or mislabeled products. Its rapid-onset, full-spectrum opioid-like withdrawal highlights the need for clinicians to recognize MP’s unique pharmacology, the limitations of standard toxicology testing, and the evolving landscape of manipulated “kratom” products. Incorporating MP into the differential diagnosis for severe or early-onset withdrawal in patients reporting kratom use is essential. Continued case reporting will help inform clinical practice, enhance surveillance of emerging products, and guide harm-reduction and public health responses.
